# The Effect of Alkyl Chain Length on Biofunction of Dietary Lipid

**DOI:** 10.3390/molecules31050841

**Published:** 2026-03-03

**Authors:** Wen-Hui Sun, Sha Liu, Wen Dai, Chin-Ping Tan, Yong-Jiang Xu

**Affiliations:** 1 State Key Laboratory of Food Science and Resources, School of Food Science and Technology, National Engineering Research Center for Functional Food, Jiangnan University, Wuxi 214122, China; sunwenhuiac@163.com (W.-H.S.); 6230111017@stu.jiangnan.edu.cn (W.D.); 2 Wuxi Hospital of Traditional Chinese Medicine, Wuxi 214071, China; ls1093@126.com; 3 Department of Food Technology, Faculty of Food Science and Technology, University Putra Malaysia, Selangor 410500, Malaysia; tancp@upm.edu.my

**Keywords:** alkyl chain length, lipids, biological function

## Abstract

Dietary lipids not only enhance the flavor and nutritional value of food, but more importantly, they offer essential fatty acids and energy for metabolism. The importance of lipid unsaturation has gained increasing attention; however, the impact of the alky chain length on biofunction of dietary lipids remains unclear. This article discusses the effects of the alkyl chain length on the biological function of lipids, focusing on physical and chemical properties, digestion and absorption, and nutritional functions. Firstly, with the increase in the chain length, the melting point of the crystal increases, the symmetry increases, and the hypersensitivity induction decreases. Secondly, the alkyl chain length affects the contact between lipid droplets and lipase, as well as the fatty acids release rate. Finally, medium-chain and short-chain lipids can partially reverse the effect of long-chain lipids. Understanding the effect of the alkyl chain length on the biofunction of dietary lipids can provide valuable insights for designing nutritious diet.

## 1. Introduction

Dietary lipids confer specific functional, nutritional, and sensory properties to food. They supply energy for metabolism, participate in cell membrane composition, control intercellular and intracellular communication, and regulate gene expression that can trigger complex cascade reactions, thereby maintaining body homeostasis [1,2,3]. Dietary lipids primarily consist of triglycerides and lipoids (phospholipids, sterols). Triglycerides, which are the main components of dietary lipid, exist in various forms, such as free (oil), droplet (emulsion), or crystallization network (butter). Upon ingestion, triglycerides are first degraded into diglycerides with the assistance of gastric lipases. Subsequently, they are further digested into monoglycerides and fatty acids by various lipase [4]. These digestive products are absorbed and metabolized to provide energy. Beyond providing energy, triglycerides can also serve as bioactive substances or auxiliaries that help ameliorate metabolic syndrome, such as corpulency and insulin resistance [5,6]. Most nutritional molecules typically exert their effects by acting on membrane proteins or intracellular targets [7], necessitating passage through the cell membrane. Modification of triglyceride can facilitate better interactions between these biomolecules and cell membranes.

In the field of dietary triglycerides nutrition, the importance of lipid unsaturation has increasingly garnered attention. Several excellent reviews on lipid unsaturation have been published in recent years [8,9,10,11]. Saturated triglycerides (SATs) are less prone to oxidation; however, they cause a greater increase in insulin resistance, endotoxemia and multiple plasma ceramides compared to unsaturated triglycerides (UNSATs) [12]. Saturated triglyceride is more likely to have a negative impact on human liver metabolism than UNSAT. Additionally, the polyunsaturated structure of PUFAs complicates the spatial conformation of their glycerides, making them resistant to hydrolysis by lipases. Next, the digestive environment accelerates the oxidation of PUFAs, leading to a further reduction in their bioavailability [13].

Indeed, numerous studies have reported the chain length of the alkyl group is another crucial factor affecting the biofunction of dietary triglycerides. Triglycerides are composed of glycerol and fatty acids, and the category of fatty acids determines the category of triglycerides. Johannes et al. [14] coupled small molecules with triglycerides to explore the biological activity and cell permeability. The results indicated that compared to long-chain triglyceride (LCT, carbon number ≥ 14) conjugates, medium-chain triglyceride (MCT, carbon number 6–12) conjugates showed better uptake and biological activity, with effects comparable to those of short-chain triglyceride (SCT, carbon number < 6) conjugates. Zhang et al. found that under the intervention of dietary lipid for atherosclerosis, the aortic sinus plaque and the entire aorta acreage were remarkably smaller in the octanoic acid group compared to the decanoic acid and stearic acid groups [15]. Furthermore, another study revealed that the effect of palmitoleic acid was stronger than that of oleic acid in improving human endothelial cell inflammation [16]. These results appear to be mediated, at least in part, by inhibiting NF-κB gene expression and up-regulating PPAR-α.

However, no comprehensive in-depth reviews on the impact of the alkyl chain length have been published. The article aims to review the functions of the carbon chain length on the biofunction of dietary triglyceride. The roles of the chain length on physical and chemical properties, digestion, and absorption are also discussed. This review serves as a reference for developing a reasonable dietary ratio to prevent and treat intestinal and metabolic diseases.

## 2. The Role of Alkyl Length in Characteristics of Triglycerides

**Figure 1:**
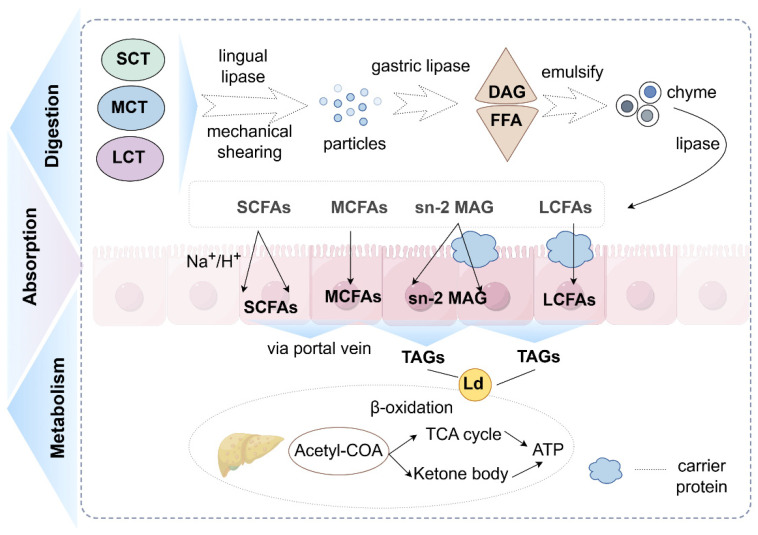
Digestion, absorption and metabolism of triglycerides (created with Figdraw). (1) Triglycerides are degraded into monoglycerides and fatty acids by a series of enzymes. (2) Fatty acids enter intestinal epithelial cells in a variety of ways (SCFAs: depend on ion carriers and diffusion; MCFAs: diffusion effect; LCFAs: protein assistance). (3) They are metabolized in the liver (SCFAs and MCFAs: direct oxidation and providing a small amount of energy; LCFAs: oxidation after re-esterification and offering most of the energy required by the body). Abbreviations: SCT, short-chain triglycerides; MCT, medium-chain triglycerides; LCT, long-chain triglycerides; DAG, diacylglycerol; FFA, free fatty acids; Ld, lipid droplet; SCFAs, short-chain fatty acids; MCFAs, medium-chain fatty acids; LCFAs, long-chain fatty acids; sn-2 MAG, sn-2 mono-acylglycerol; TAGs, triacylglycerols; TCA cycle, tricarboxylic acid cycle; Acetyl-CoA, acetyl coen-zyme A.

Triglycerides exhibit diverse structures and abundant contents in nature. Additionally, they have multiple metabolic pathways influenced by the alkyl length. Therefore, understanding the digestion, absorption, and metabolism of different triglycerides can help us better grasp the impact of alkyl chain length on the biological function of triglycerides and fatty acids (Figure 1).

### 2.1. Origin

Triglycerides are naturally present in multiple foods, e.g., meat, plants, nuts, and milk [17]. The primary plant sources of SCTs and MCTs are coconut oil and palm kernel oil [18,19]. Meanwhile, they are widely found in milk powder, especially in premature infant formula, where the content of medium-chain triglyceride can reach up to 50% of fat content [20,21]. Medium- and long-chain triacylglycerol (MLCT) are primarily found in mammalian milk fat, such as breast milk, goat milk, and related products, and have high nutritional value [22,23]. Ruminant milk fat contains short-chain fatty acids (SCFAs, carbon number < 6), including butyric acid (10 mol%), caproic acid (5 mol%), and acetic acid [24]. Short-chain fatty acids can also be generated by the fermentation of dietary fiber by intestinal anaerobic microorganisms [25]. For instance, *Prevotella* is a strong producer of SCFAs, primarily generating acetic acid [26]. Butyric acid is produced by the fermentation of *Fecal bacilli* [27]. Additionally, valeric acid is mainly produced by *Megasphaera massiliensis* [28]. Natural sources of medium-chain fatty acids (MCFAs, carbon number 6–12) include breast milk, where the uptake is about 15% of total fatty acids [29]; the content in mature milk was the highest, being about 42.18% higher than that in transition milk [30]. The quantity of MCFA in term milk was significantly higher than that in preterm milk [30]. Moreover, octanoic acid and lauric acid can also be derived from goat milk [31,32]. Long-chain fatty acids (LCFAs, carbon number ≥ 14), including long-chain saturated fatty acids (LCSFAs) and long-chain unsaturated fatty acids (LCUFAs), are widely found in various fats, in particular, vegetable oil, soybean oil, peanut oil, rapeseed oil, and animal lard and butter.

### 2.2. The Impacts on Physicochemical Properties

The alkyl chain length affects the thermal properties and polymorphism of aliphatic acids and esters. For saturated straight-chain aliphatic acids and esters, the melting point and heat capacity increase with the even number of the alkyl chain length, but the difference in the upward trend between the two is not significant. Moreover, the melting point of whole triglycerides is higher than that of free fatty acids [33,34]. However, the trend is not evident for unsaturated aliphatic acids, which are mainly influenced by the configuration and position of double bonds. Meanwhile, thermal conductivity and chemical structure do not change significantly with the chain length. Hagemann et al. revealed the stability of the β-form increased as the acyl chains extended from 16 to 22 carbons. In a recent study on the functions of triacylglycerol components in the crystallization behavior of milk fat [35], Yoga et al. confirmed that fully saturated LCTs were beneficial to the production of 2L crystals and triglycerides containing SCFAs, which conduce to the formation of 3L crystals. Moreover, triglycerides containing SCFAs restrained the crystallization process of milk fat to a certain extent. Furthermore, fatty acids can also combine with carbohydrates, such as starch, to form nanocomposite materials. As the chain length increased from C12 to C22, the encapsulation efficiency of fatty acids improved, and the compound’s emulsification enhanced [36].

The chain length of fatty acids affects the function of biological membranes. Long-chain fatty acids (e.g., C18–C28) enhance intermolecular van der Waals forces between lipid hydrocarbon tails, leading to a higher phase transition temperature (Tm) of the lipid bilayer [37]. This stronger intermolecular interaction reduces the lateral diffusion capacity of lipid molecules and membrane proteins, thereby decreasing membrane fluidity and rendering the membrane at a more rigid gel state. In contrast, short-chain fatty acids (e.g., C12–C16) have weaker intermolecular hydrophobic interactions due to their shorter hydrocarbon tails, resulting in a lower Tm and looser lipid packing; this loose arrangement increases the mobility of lipid molecules, improves membrane fluidity, and maintains the membrane in a dynamic liquid–crystalline state that facilitates material transport and signal transduction. Lipid packing density in the biological membrane is also closely associated with the fatty acid chain length. Long-chain fatty acids can extend deeper into the hydrophobic core of the lipid bilayer, reducing the gaps between adjacent lipid molecules and forming a more ordered and denser packing structure; this dense packing not only enhances the mechanical stability of the membrane but also limits the penetration of small molecules and ions across the membrane [38]. On the contrary, short-chain fatty acids have limited extension in the hydrophobic core, resulting in larger gaps between lipid molecules and relatively loose packing; such loose packing may increase the permeability of the membrane to small hydrophobic molecules while reducing the membrane’s resistance to external mechanical stress. Notably, the effect of the fatty acid chain length on lipid packing can further regulate the formation of lipid domains (e.g., raft domains) in the membrane, which are crucial for the localization and function of membrane proteins [39]. Collectively, the chain length of fatty acids incorporated into biological membranes serves as a key structural factor that modulates membrane biophysical properties and further mediates membrane functionality, laying a foundation for understanding the structure–bioactivity relationship.

In summary, the chain length is a crucial factor in the physicochemical properties of fatty acids, triglycerides, and other related compounds.

### 2.3. The Impacts on Digestion and Absorption Behaviors

Triglycerides are hydrophobic molecules, and their apolarity prevents them from being transported directly from the lumen of gut to the intestinal epithelium. Triglycerides are initially digested by the mouth, stomach, and intestine, and eventually form monoglycerides and free fatty acids. Triglyceride digestion typically involves three steps: first, triglycerides are broken down into smaller particles through mechanical shearing, such as chewing, swallowing, and hydrolysis of lingual lipase in the mouth; second, diacylglycerol and remaining triglycerides are further emulsified; and finally, various lipase act to hydrolyze fats into monoacylglycerol and free fatty acids. Various enzymes are involved in the digestion of dietary triglycerides within the body. Fatty acids are initially released by hydrolysis from Sn-1 and Sn-3 positions of triglycerides, while Sn-2 monoglyceride is retained to fulfill its critical nutritional and metabolic roles [40].

The chain length of alkyl is closely related to triacylglycerol digestion. As chain length increases, the degradation rate of triacylglycerol decreases. Previous research has indicated that short-chain and medium-chain triacylglycerol have higher rates of hydrolysis and metabolism when compared with long-chain triacylglycerol [41]. These results may be closely associated with the structure of triglyceride and its various digestive enzymes.

Short-chain triglycerides (SCTs) and medium-chain triglycerides (MCTs) They are more easily digested, which may be ascribed to the hydrolyzation characteristics of lipase. Compared to LCFAs, gastric lipase prefers to hydrolyze SCFAs and MCFAs at the Sn-3 position, while pancreatic lipase is more efficient in hydrolysis of Sn-1,3 MCFAs [17]. Moreover, short-chain triglycerides (SCTs) form smaller droplets when emulsified in the stomach, thereby increasing the surface area bound to lipase. Early in life, the content of pancreatic lipase and bile salts is limited in the body. Consequently, tongue and stomach lipases exist a necessary role in the decomposition of triglycerides. Lingual lipase has a higher affinity to MCTs than for LCTs [3]. Tongue lipase, secreted by the tongue gland along with the food from the mouth, hydrolyzes Sn-3 fatty acids in the stomach [42]. An in vitro study demonstrated that the activity of lingual lipase’s activity towards tricaprylate is 10 times greater than towards triolein. An in vivo study showed the digestion rate of LCT is lower than that of MCT in preterm infants, primarily due to the utilization of alternative digestion pathways involving in lingual and gastric lipase [43]. During digestion, the released LCFAs tend to accumulate at the oil–water boundary, significantly reducing the rivalrous absorption of bile salts, and limiting pancreatic lipase’s contact with fat droplets. Short-chain fatty acids and medium-chain fatty acids exhibit higher affinity for water, enabling them to rapidly enter the surrounding aqueous phase. This provides additional absorption sites at the oil–water interface of lipid droplets, facilitating easier access to the lipid surface for pancreatic lipases [44].

Triglycerides are primarily absorbed by the small bowel and oxidized in the liver to provide energy [45]. The length of the alkyl chain affects the absorption and metabolism of fatty acids and monoglycerides. It influences the absorption of substances through three mechanisms: intestinal cell absorption, intracellular processing, and mesenteric lymph reception [46]. McKimmie et al. found that the FFA absorption efficacy rate decreased with the increase in chain length, from 0.953 ± 0.017 for tetradecanoic acid to 0.798 ± 0.032 for octadecanoic acid, and extremely dropped to 0.264 ± 0.02 for eicosanoic acid [47]. This result can, at least in part, be attributed to the differing absorption pathways of multiple fatty acids and monoglycerides. Short-chain fatty acids enter enterocyte through four mechanisms [48]: a nonionized proton dispersion form, when converted to SCFA-2HCO_3_^−^, a hydrogen-coupled monocarboxylate transporter, and as a sodium-coupled monocarboxylate transporter. Medium-chain fatty acids are absorbed through passive diffusion due to their small molecular weight and strong water solubility [49]. Long-chain fatty acids are transported into colonic epithelial cells by binding with carrier proteins, such as fatty acid binding proteins (FABP) and fatty acid transport proteins (FATP) [50,51,52]. Additionally, long-chain fatty acids obtained through digestion may precipitate into fatty acid calcium soaps and form crystals at body temperature, thereby affecting the absorption of fatty acids. Monoglycerides and fatty acids are transported to the endoplasmic reticulum (ER) and synthesized into triglycerides again. They then form lipid droplets for energy storage in cells. However, medium-chain fatty acids exhibit low affinity with lipid anabolic enzymes (e.g., diglyceride acyltransferase) [18], which facilitate rapid oxidation. Diglyceride acyltransferase mainly exists in the endoplasmic reticulum (ER) and lipid droplets. It attaches to the superficies of lipid droplets through the C-terminal amphiphilic α-helix, thereby regulating fat absorption, triacylglycerol resynthesis and cholesterol metabolism [53]. Following intracellular processing, substances enter the *lamina propria* via exocytosis and ultimately reach the mesenteric lymph node.

Digested fatty acids are transported in various ways in the body. Short-chain fatty acids that enter into the systemic circulation by means of the *vena portae* are then delivered to the peripheral tissues for oxidation and energy supplying [54]. Medium-chain fatty acids, the main ketogenic fatty acids, are directly circulated through the portal vein and rapidly metabolized in the body [55]. Additionally, they can be transported as free fatty acids or connected to plasma albumin [56]. Long-chain fatty acids must be re-esterified in the small bowel to shape chylomicrons, which are then transported via lymph vessels and blood vessels [57]. The conjugation of aliphatic acid to serum albumin influences their transportation, thereby affecting the body’s uptake. Kragh-Hansen et al. [58] proved that the primary association constants of aliphatic acid with serum albumin increase with the chain length, but not linearly. The ratio between K1 for decanoic acid/caprylic acid is 2.6 and the K1 of laurate is 6.7 times that of decanoate. Besides that, the quantity and location of high-intimacy binding sites in the albumin molecular are likely determined by the chain length [59,60]. In addition, studies indicated that the macromolecule crowding affects the combination of aliphatic acid to serum albumin [56]; in a crowded nutrient environment, the configuration of BSA is loosened, promoting the binding of MCFA–BSA.

Regarding oxidative metabolism, butyrate is superior to ethane acid, propionic acid, and even glutamine, gluconate, and acetone bodies [61]. It serves as a crucial respiratory fuel for the body. Simultaneously, medium-chain fatty acids are preferentially metabolized to produce energy compared to LCFAs in the liver [62]. Long-chain fatty acids exhibit relatively slow metabolic rates and store a certain amount in tissues, and their oxidation is partially dependent on carnitine [63,64].

From the above, the alkyl chain length of triglycerides affects their digestion, absorption transport and metabolism. The given metabolic behavior of triglyceride is well linked to their profitable physiological function in vivo. Therefore, the alkyl chain length can be used as an important factor to optimize the proportion of fatty acids and enhance dietary nutrition.

## 3. The Role on Nutritional Properties

**Figure 2:**
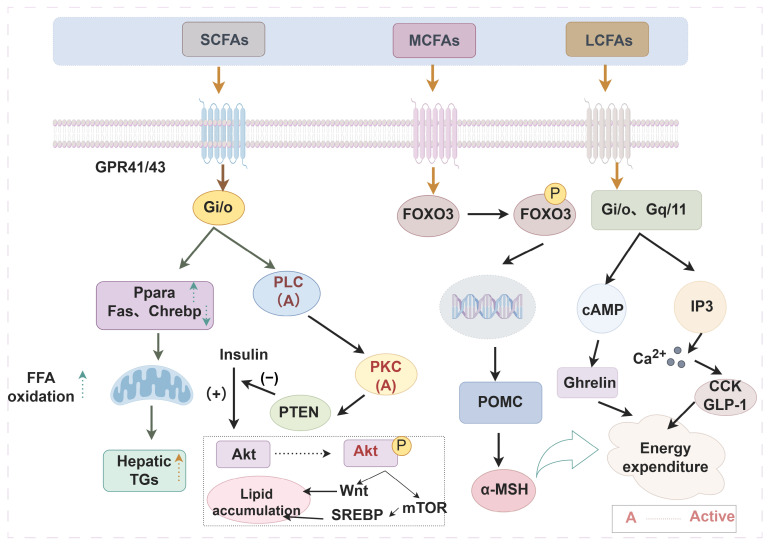
Effects of different fatty acids on peripheral tissue metabolism or function (By Figdraw) The alkyl chain length affects the biological function of fatty acids through multiple mechanisms. Take regulating obesity as an example, SCFAs act on GPR41 and GPR43 to interfere with downstream gene expression, thereby promoting lipid metabolism. MCFAs and LCFAs apply to GPR40 and GPR120, respectively, regulate transcription factor expression and hormone secretion, and control energy metabolism, thus enhancing satiety. Arrows outside the text boxes indicate the subsequent induced reaction, while arrows inside the text boxes represent a trend of change. ↑—up-regulation; ↓—down-regulation. Abbreviations: SCFAs, short-chain fatty acids; MCFAs, medium-chain fatty acids; LCFAs, long-chain fatty acids; Gi/o, guanine nucleotide-binding protein, inhibitory α subunit; PLC, phospholipase C; PKC, protein kinase C, PTEN, phosphatase-tensin gene; SREBP, sterol-regulatory element binding protein; POMC, pro-opiomelanocortin; CCK, cholecystokinin; GLP-1, glucagon-like peptide-1; Ppara, peroxisome proliferator-activated receptor alpha; FFA, free fatty acids; TGs, triglycerides; Akt, ak strain transforming; Fas, fatty acid synthase; Chrebp, carbohydrate response element binding protein; mTOR, mammalian target of rapamycin; FOXO3, forkhead box protein O3; Gq/11, guanine nucleotide-binding protein, q/11 polypeptide; α-MSH, α-melanocyte-stimulating hormone; Ghrelin, growth hormone secretagogue.

To date, numerous studies have been carried out on the intestinal health changes induced by dietary triglycerides and fatty acids (Table 1). Moreover, they can influence physiological state in vivo by acting on certain molecules or specific proteins (Figure 2). In series of studies, we observed that the alkyl chain can affect the nutritive peculiarities of fatty acids and triglycerides. These circumstances will be addressed below.

### 3.1. Effects on Intestinal Microenvironment

Different types of triglycerides regulate the intestinal microenvironment in varied ways. The study by Kripke et al. [74] investigated the impacts of dietary intervention on gut health in short bowel syndrome (SBS). It was found that compared to the supplemental chemical definition (CD) group and the MCT diet group, the diet supplemented with SCT lead to a significant increase in mucosal weight, intestinal segment weight, mucosal protein and DNA expression level of jejunum and colon. Moreover, short-chain triglycerides promoted the adaptive growth in the jejunum and colon in SBS and maintained a comparable nutritional status. They ameliorated metabolic syndrome and maintained the intestinal microenvironment by multiple mechanisms. Studies have shown that butyrate glycerides not only abated the immune-inflammatory response by restraining the NF-κB/MAPK pathways but modulated the intestinal bacteria to improve intestinal health [72]. Meanwhile, valeric acid glycerides (GVA) obviously raised the proportion of the villus height to crypt depth in the jejunum and the density of enteroendocrine cells producing glucagon-like peptide-2 in order to diminish the occurrence rate of necrotic enteritis [69]. Tricaprylin mainly maintained intestinal morphology by improving digestive enzyme activity, the concentration of SCFAs, and the secretion of protein related to intestinal permeability, while reducing the inflammatory cytokine levels and the abundance of hazardous intestinal microbiota [73].

Fatty acids can influence the intestinal microenvironment. Infusing LCFAs into the ileum of humans and rats slowed small bowel transportation time. In contrast, short-chain fatty acids accelerated transport via a topical enteric reflex. Ethane acid (20 mM, 50 mM, 100 mM), butanoic acid (100 mM), and octoic acid (100 mM) significantly accelerated migration. The transportation velocity was inversely proportional to SCFA carbon chain length. Moreover, the colon can easily absorb these SCFAs, recovering some of the lost energy [75]. Short-chain fatty acids can promote intestinal impermeability and improve lipidic metabolism, lessening the occurrence of metabolic syndromes such as obesity [66,67]. They can also adjust the secretion of antifungal peptides from the intestinal epithelial cells. They can stimulate RegIIIγand defensins generation by reactivating mTOR and STAT3, thus strengthening the defense capability of intestinal epithelial barrier [68]. It was found that adding acetate to the growth medium could boost the level of histone acetylation in *Salmonella enterica* and *Bacteria coli* [37,76]. The acetylation of PhoP protein cut down the binding capacity of PhoP protein to DNA, thus weakening the toxicity of Salmonella. Butyrate inhibited the accumulation of enteric pernicious bacteria while accelerated the colonization of conducive bacteria, such as Actinobacteria, *Bifidobacterium bifidum*, and Bacteroidetes [77]. Butyrate also coordinated the gut barrier protection by improving epithelial O_2_ consumption which is conducive to the stability of transcription factor, such as hypoxia inducible factor (HIF) [66]. Medium-chain fatty acids have gained increasing attention owing to their possibly favorable antimicrobial effect. There were studies that proved MCFs were involved with a wide range of intestinal microflorae. They exerted protective effects by decreasing part of the colonization of the gut microbiome, including *Campylobacter*, *Clostridium*, *Salmonella*, and *Escherichia coli*, with an average of 44%, 78%, 79%, and 66%, respectively, thus promoting the nutritional and health status of poultry and decreasing the risk of increased antibiotic resistance in animals [70]. Lauric acid can be a naturally useful substance in restraining *Clostridioides difficile* growth. The generation of reactive oxygen species (ROS) and cell membrane lesions are part of the mechanism by which lauric acid can play a role [78]. Gastrointestinal function is closely related to the enteric nervous system, and the intestinal microenvironment can be maintained through the brain–gut axis. Palmitic acid (PA) caused an evident absence of myenteric neurons in both the ileum and colon by mediating the ADP-sensitive *P2y13* receptor, thereby impairing intestinal health [65]. However, palmitoleic acid also increased the abundance of *Akkermansia muciniphila*, changed the content of SCFAs in the intestine, and ameliorated intestinal inflammation [79]. The increase in the carbon chain length of fatty acids follows the fundamental rules of their biosynthesis. In the classical de novo synthesis pathway catalyzed by fatty acid synthase, each elongation cycle uses malonyl-CoA as the donor of the two-carbon unit, resulting in the vast majority of natural fatty acids having an even number of carbon atoms. Despite exceptions, odd-chain fatty acids (e.g., pentadecanoic acid, heptadecanoic acid) synthesized using propionyl-CoA as the initiator can be of low abundance in nature, primarily derived from branched-chain amino acid metabolism, intestinal flora fermentation, and ruminant fat. Therefore, this study focused primarily on the properties of even-chain fatty acids.

### 3.2. Effects on Peripheral Tissue Metabolism or Function

The alkyl chain length changes the nutritional efficacy of triglycerides. In a double-blind randomized crossover research, sufferers with Crohn’s disease or bowel bypass syndrome were treated with triacylglycerol emulsion for four weeks. The results manifested when two of those treated with LCT emulsions exhibited aberrant liver function, which was then consequently reversed when switching to a structured triglyceride therapy. Hence, structured triglycerides including medium- and long-chain fatty acids may be relevant with a possible reduction in liver dysfunction [80]. In addition, supplementing MCTs in dietary LCTs can amplify postprandial caloric production. Research has indicated that MCTs can exert a key role in obesity management by increasing energy consumption and satiety [81,82]. This effect of MCTs may be related to brown fat (BAT) activation in animals [5]. When the presentation of β3_adrenergic receptor (β3_AR) protein and uncoupling protein 1 (UCP1) up-regulated in BAT and the noradrenaline (NE) pathway was activated, this can trigger many biochemical lipolysis reactions [83]. Furthermore, when there exists a lot of glycogen in vivo, MCTs can also undergo lipid metabolism to generate ketone B-hydroxy butyric acid (BHBA). B-hydroxy butyric acid can affect brain activity through the blood–brain barrier, regulat nerve center signals related to food intake, reduce appetite, and even produce anorexia [84]. Long-term consumption of LCTs may pose health risks, but moderate intake of LCTs is essential in daily life. This is because LCTs serve as the primary dietary source of essential fatty acids (linoleic acid and alpha-linolenic acid). Furthermore, LCTs act as vital carriers for the absorption and transport of fat-soluble vitamins (A, D, E, K).

The effects of dietary fatty acids on metabolism or on the function of peripheral tissues are different. Exploring the effects of SCFAs on adipose degeneration and inflammation in metabolic dysfunction-associated steatohepatitis (MASH) mice, three main SCFAs (acetate, propionate, and butyrate) were selected for intervention. The results showed that SCFAs could alleviate MASH, among which sodium acetate had the best effect [85]. The mechanism of action was to affect AMPK activation and inhibit macrophage pro-inflammatory differentiation. In human endothelial cells, studies have displayed that palmitoleic acid has better anti-inflammatory effects than oleic acid and palmitic acid, mainly by reducing the expression of NF-κ B and the release of inflammatory factors, up-regulating the presentation of the PPAR-α gene, and preventing the occurrence of endothelial dysfunction [16].

Diverse fatty acids can act on different fatty acid receptors, carry out signal transduction through various mechanisms, and regulate body metabolism. The fatty acid receptor FFAR2/3 can exhibit a common motif that SCFA can recognize. There is a PAM binding pocket in FFAR3. At the same time, AR420626 (selective agonist of FFAR3/GPR41) can interact with Gαi to provide a targeted receptor–transduction interface for the recognition of fatty acids with different carbon chain lengths [86]. Short-chain fatty acids could mediate the free fatty acid receptor FFAR43 and regulate the transcript of lipid metabolism genes (*Fas*, *Ppara*, *Chrebp*, etc.) in the liver, playing an anti-obesity role [87]. Acetate-mediated GPR43 activation can inhibit insulin-induced Akt phosphorylation through the G (i/o) βγ-PLC (phospholipase C)-PKC (protein kinase C)-*PTEN* (phosphatase-tensin gene) signaling pathway [88]. Inhibiting insulin biological activity can promote energy consumption, accelerate lipid burning in adipose tissue, and avoid lipid accumulation. Acetate could also regulate instantaneous contraction and control blood pressure through direct target receptor GPR43 [89]. Propionate can reduce blood pressure by mediating the activation of olfactory receptor 78 (olfr78) and G protein-coupled receptor 41 (GPR41) [45]. Propionate can also alleviate myocardial I/R injury aggravated by Angiotensin II dependent on caveolin-1/ACE2 (angiotensin-converting enzyme 2) axis through GPR41 [90]. Octanoic acid can control food intake and prevent various complications caused by obesity [91]. The mechanism mainly for this was that it selectively activated the excitability of pro-opiomelanocortin (POMC) neurons through GPR40 and stimulated changes in energy status via indirect non-synaptic, purines, and adenosine receptor-dependent mechanisms. Such changes were not observed in the oleic acid treatment group. Octanoic acid was also associated with the activation of GPR84, affecting downstream signal transduction and preventing disease [92]. G protein-coupled receptor 120 (GPR120, FFAR4) is a LCFA sensor, mainly long-chain unsaturated fatty acids (LCUFAs) [93]. LCUFAs lured GLP-1 secretion from STC-1 endocrine c’s of gut cells to control appetite and enhance sensitivity in a FFA4-dependent manner [94]. In addition, GPR120 mRNA was enriched in mice and human microglia; acting on this target contributed to neuroimmunomodulation and behavior improvement [95].

## 4. Conclusions and Prospects

Short- and medium-chain fatty acids (SCFAs/MCFAs) exhibit distinct functional advantages. Their shorter chains facilitate higher membrane fluidity and more efficient interaction with metabolic enzymes (e.g., lipases), leading to rapid absorption and oxidation. These properties underpin their therapeutic and nutraceutical potential in mitigating diet-induced obesity and metabolic disorders. In contrast, many LCFAs, such as palmitic acid, are often associated with pro-inflammatory responses and adverse metabolic effects, highlighting the critical importance of the chain length in functional outcomes.

However, the biological activity of lipids does not increase linearly with the chain length; it often diminishes beyond an optimal point, a phenomenon requiring further mechanistic exploration. Future research should move beyond one-dimensional analyses of the chain length. We propose establishing a multi-dimensional evaluation system that integrates the chain length, degree of unsaturation, and double-bond position to comprehensively predict lipid functionality. Additionally, investigating the synergistic interactions between designed lipids and other dietary components (proteins, carbohydrates) will be crucial for developing effective nutritional strategies to combat metabolic diseases.

## Figures and Tables

**Table 1:** Effects of fatty acids and triglycerides on intestinal microenvironment.

Study Model	Lipid Information	Finding	References
P2Y13 +/+ and P2Y13 −/− mice	PA (Sigma-Aldrich, St. Louis, MO, USA)	By mediating the ADP-sensitive *P2y13* receptor, activation of intestinal neuronal apoptosis ↑	[65]
HIF-luciferase reporter mice and caco-2 cells	Sodium butyrate (10 mM)	HDAC inhibitor made HIF change the transcription of genes that could not be obtained by chromatin structure. HIF ↑	[66]
Animal ang cell experiments	Dietary medium-chain triglycerides (MCT)	Bifidobacteria ↑, Gram-negative bacteria ↓, the content of SCFAs ↑	[67]
C57BL/6 mice, GPR43−/− mice	Butyrate or acetate (300 mM)	RegIIIγ and β-defensins 1, 3, and 4 ↑	[68]
Ross-308 chicks	Valeric acid glycerides (GVA)	Body weight ↑, feed conversion rate ↓, the density of l cells that produce GLP-2 ↑	[69]
In poultry (chicken intestines)	MCFAs	Campylobacter, Clostridium Salmonella and Escherichia coli ↓	[70]
Half of the mice received FABP4 inhibitor BMS309403 (1 mg/kg; twice a week)	STD with 7% (by weight) coconut oil (COCO) or with 7% evening primrose oil (EPO).	Compared to STD, COCO: GI transit ↑, but not colonic motility; EPO: colorectal distension (CRD) ↑	[71]
In piglets challenged with ETEC	0.5% glyceryl butyrate	*L. reuteri*, *Bifidobacterium longum*, *Lactobacillus* sp. ↑, IL-1β and THF-α ↓	[72]
C57BL/6 mice, seven-week-old	The normal diet incorporating 2% tricaprylin	*Lactococcus*, *Enterococcus*, and *Roseburia* ↓, and reduced TNF-α and IL-1β ↓, claudin-1 and ZO-1 and the concentration of SCFAs ↑	[73]
Mouse CRC model	2% butyrate salts (Sigma Chemical Co., St. Louis, MO, USA)	*Lactobacillales* and *Enterobacterales* ↓	[23]

Note: ↑—improve; ↓—decrease. MCFAs, medium-chain fatty acids; STD, standard diet; GI, gastrointestinal; SCFAs, short-chain fatty acids.

## Data Availability

No new data were created or analyzed in this study.
